# Integrated Sustainable childhood Pneumonia and Infectious disease Reduction in Nigeria (INSPIRING) through whole system strengthening in Jigawa, Nigeria: study protocol for a cluster randomised controlled trial

**DOI:** 10.1186/s13063-021-05859-5

**Published:** 2022-01-31

**Authors:** Carina King, Rochelle Ann Burgess, Ayobami A. Bakare, Funmilayo Shittu, Julius Salako, Damola Bakare, Obioma C. Uchendu, Agnese Iuliano, Adamu Isah, Osebi Adams, Ibrahim Haruna, Abdullahi Magama, Tahlil Ahmed, Samy Ahmar, Christine Cassar, Paula Valentine, Temitayo Folorunso Olowookere, Matthew MacCalla, Hamish R. Graham, Eric D. McCollum, Adegoke G. Falade, Tim Colbourn, on behalf of the INSPIRING Project Consortium, Carina King, Tim Colbourn, Rochelle Ann Burgess, Agnese Iuliano, Hamish R. Graham, Eric D. McCollum, Tahlil Ahmed, Samy Ahmar, Christine Cassar, Paula Valentine, Adamu Isah, Adams Osebi, Ibrahim Haruna, Abdullahi Magama, Ibrahim Seriki, Temitayo Folorunso Olowookere, Matt McCalla, Adegoke G. Falade, Ayobami Adebayo Bakare, Obioma Uchendu, Julius Salako, Funmilayo Shittu, Damola Bakare, Omotayo Olojede

**Affiliations:** 1 Department of Global Public Health, Karolinska Institutet, Tomtebodavägen 18, 171 65 Stockholm, Sweden; 2 Institute for Global Health, University College London, London, UK; 3 Department of Community Medicine, University College Hospital, Ibadan, Nigeria; 4 Department of Paediatrics, University of Ibadan, Ibadan, Nigeria; 5 Department of Community Medicine, University of Ibadan, Ibadan, Nigeria; 6 Save the Children International, Abuja, Nigeria; 7 Save the Children UK, London, UK; 8 GlaxoSmithKline (GSK), Lagos, Nigeria; 9 GlaxoSmithKline (GSK), Brentford, UK; 10 Department of Paediatrics, University College Hospital, Ibadan, Nigeria; 11 Centre for International Child Health, Murdoch Children’s Research Institute, University of Melbourne, Royal Children’s Hospital, Parkville, Victoria Australia; 12 Eudowood Division of Pediatric Respiratory Sciences, Department of Pediatrics, School of Medicine, Johns Hopkins University, Baltimore, USA

**Keywords:** Child mortality, Cluster randomised controlled trial, Participatory learning and action, Community, Pneumonia, Nigeria

## Abstract

**Background:**

Child mortality remains unacceptably high, with Northern Nigeria reporting some of the highest rates globally (e.g. 192/1000 live births in Jigawa State). Coverage of key protect and prevent interventions, such as vaccination and clean cooking fuel use, is low. Additionally, knowledge, care-seeking and health system factors are poor. Therefore, a whole systems approach is needed for sustainable reductions in child mortality.

**Methods:**

This is a cluster randomised controlled trial, with integrated process and economic evaluations, conducted from January 2021 to September 2022. The trial will be conducted in Kiyawa Local Government Area, Jigawa State, Nigeria, with an estimated population of 230,000. Clusters are defined as primary government health facility catchment areas (*n* = 33). The 33 clusters will be randomly allocated (1:1) in a public ceremony, and 32 clusters included in the impact evaluation.

The trial will evaluate a locally adapted ‘whole systems strengthening’ package of three evidence-based methods: community men’s and women’s groups, Partnership Defined Quality Scorecard and healthcare worker training, mentorship and provision of basic essential equipment and commodities. The primary outcome is mortality of children aged 7 days to 59 months. Mortality will be recorded prospectively using a cohort design, and secondary outcomes measured through baseline and endline cross-sectional surveys.

Assuming the following, we will have a minimum detectable effect size of 30%: (a) baseline mortality of 100 per 1000 livebirths, (b) 4480 compounds with 3 eligible children per compound, (c) 80% power, (d) 5% significance, (e) intra-cluster correlation of 0.007 and (f) coefficient of variance of cluster size of 0.74. Analysis will be by intention-to-treat, comparing intervention and control clusters, adjusting for compound and trial clustering.

**Discussion:**

This study will provide robust evidence of the effectiveness and cost-effectiveness of community-based participatory learning and action, with integrated health system strengthening and accountability mechanisms, to reduce child mortality. The ethnographic process evaluation will allow for a rich understanding of how the intervention works in this context. However, we encountered a key challenge in calculating the sample size, given the lack of timely and reliable mortality data and the uncertain impacts of the COVID-19 pandemic.

**Trial registration:**

ISRCTN 39213655. Registered on 11 December 2019

**Supplementary Information:**

The online version contains supplementary material available at 10.1186/s13063-021-05859-5.

## Background

Pneumonia remains the leading cause of infectious childhood deaths, with half of these deaths occurring in just 5 countries, one of which is Nigeria [1]. Northern Nigerian states account for six of the top 10 respiratory mortality hotspots in Africa, including Jigawa State [2]. However, high-quality data on pneumonia incidence, case fatality rates and the point prevalence of different pneumonia severity classifications are lacking [3]. An exploration of drivers for the high pneumonia morbidity and mortality burden in this setting found low coverage of protective and preventive factors (e.g. vaccine coverage and clean cooking) [3] and multiple misconceptions [4]. Diverse stakeholders prioritised the strengthening of primary care and community knowledge to tackle pneumonia in the context of Jigawa State [5].

Several community-based participatory empowerment approaches for improving maternal, neonatal and child health outcomes have been trialled. Those which encourage collective critical consciousness and problem solving have been effective in reducing maternal and neonatal mortality [6] and more recently in reducing diabetes [7]. Participatory Learning and Action (PLA) cycles have been adapted in several cultural contexts and are recommended by the World Health Organization (WHO) as an approach to mobilising community participation in health [8].

In parallel, increasing knowledge and awareness of pneumonia needs to be met with the ability to supply high-quality healthcare services. Issues with equipment, supplies and healthcare worker knowledge of diagnosing and treating children according to WHO’s Integrated Management of Childhood Illnesses (IMCI) guidelines were seen in Jigawa [9]. Maintaining standards of care, particularly at primary care levels, where the majority of cases present and staff have less training, is challenging. Quality improvement interventions which consider health worker motivation, supportive supervision and accountability mechanisms are needed [10]. As with the community, such problem-solving interventions go beyond passive training to facilitate, motivate and empower health workers toward improved practice. The WHO has highlighted the importance of the interface between health systems and the communities they serve, focussing on the need for shared spaces to develop and enable people-centred services [11].

The root causes of a high paediatric pneumonia burden in Jigawa are complex and multi-faceted, spanning individual, community and health system factors. Therefore, interventions to improve illness recognition, care-seeking, care provision and subsequent outcomes need to take a whole systems perspective. The aim of the ‘Integrated Sustainable childhood Pneumonia and Infectious disease Reduction in Nigeria’ (INSPIRING) trial is to evaluate if a complex intervention of community groups linked to primary care and health system strengthening will reduce child mortality and improve child health, in Jigawa State.

## Methods

**Fig. 1:**
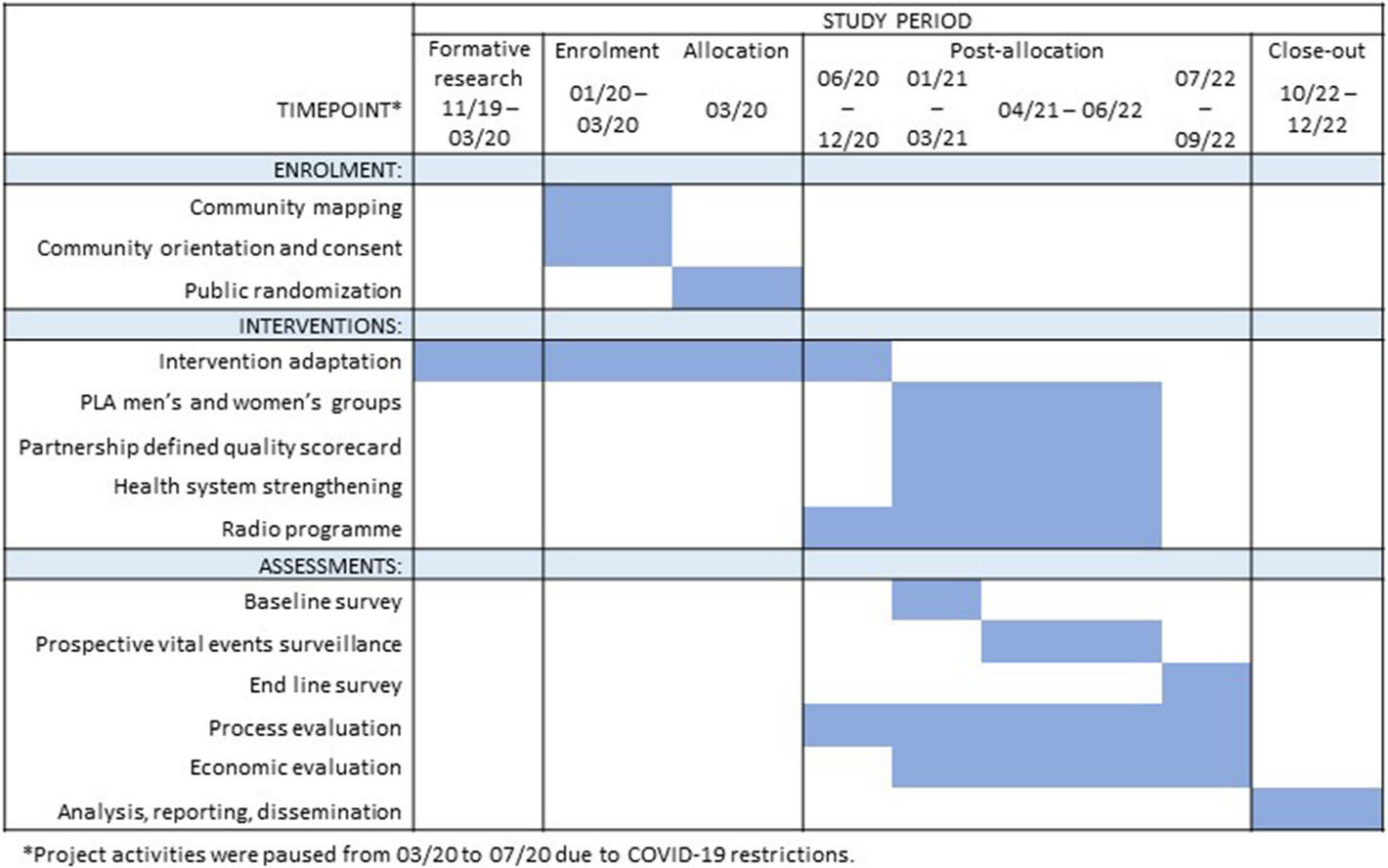
SPIRIT trial schematic

The INSPIRING Jigawa Trial is a cluster randomised controlled trial (cRCT), evaluating a three-part whole system strengthening intervention package, in Kiyawa Local Government Area (LGA), Jigawa State, Nigeria. This will be complemented with an embedded ethnographic process evaluation and economic evaluation to determine cost-effectiveness. The trial will run from January 2021 to September 2022, following a formative phase and COVID-19 adaptation from December 2019 to December 2020 (Fig. 1). The primary research question and secondary process and economic evaluation research questions are detailed in Table 1.

**Table 1:** Impact, process and economic evaluation research questions

Impact evaluation *(primary research question)*	What is the impact of a package of gender-sensitive group-based problem-solving interventions at community and community-facility levels to improve protection, prevention, diagnosis and treatment of childhood pneumonia and infectious diseases on mortality of children under-5 years old in Kiyawa LGA, Nigeria?
**Process evaluation**	Which attributes, mechanisms, effects (intended and unintended) and contextual factors enable or prevent gender-sensitive group-based problem-solving interventions at community and community-facility levels to improve protection, prevention, diagnosis and treatment of childhood pneumonia and infectious diseases in Kiyawa LGA?
	How do various stakeholders understand their role in the process of childhood pneumonia prevention in Jigawa, and how does this perception shift through participation in community-level interventions?
	How do social norms around gender, participation and decision-making in the home shape participation and engagement in community-level interventions to tackle childhood pneumonia?
	How do power and social dynamics and relationships in the wider community shape channels of communication between families and practitioners, and how does this impact on the ability to implement and efficacy of community-facility referral pathways?
	Was the intervention delivered (including fidelity, dose, reach, intensity, adaptation and duration) as intended?
**Economic evaluation**	What is the cost per disability-adjusted life-year (DALY) averted of a package of group-based problem-solving interventions at community and community-facility levels to improve protection, prevention, diagnosis and treatment of childhood pneumonia and infectious diseases in Jigawa State, Nigeria?
	Is it cost-effective considering opportunity costs of current and projected health spending (i.e. what is the estimated net benefit of the intervention package)?
	If the intervention package is cost-effective, is it affordable given the budget required for scale-up in Jigawa State, Nigeria?

### Setting

**Fig. 2:**
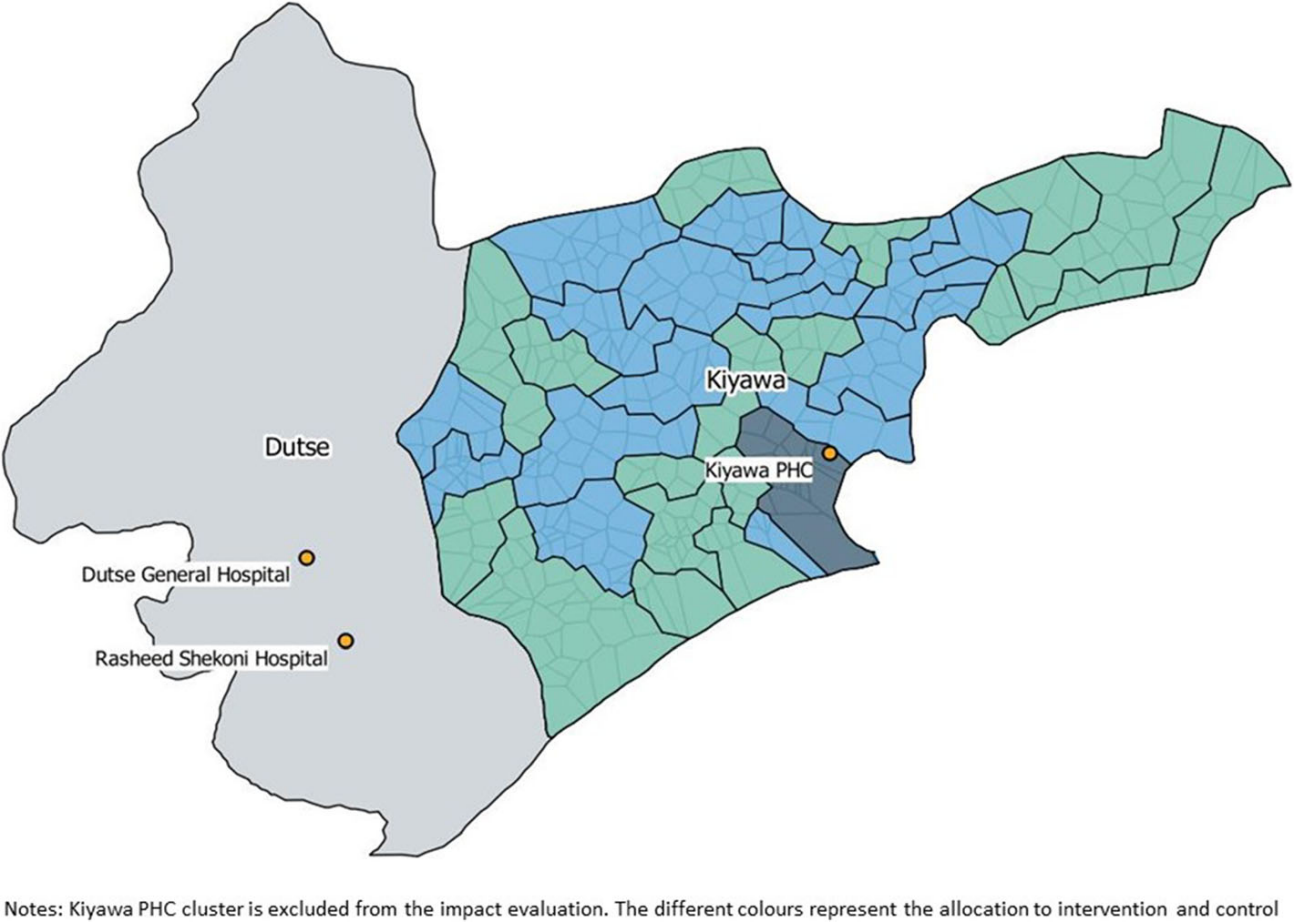
Map of Kiyawa LGA with study clusters

The trial will be conducted in Kiyawa LGA, Jigawa State. The population is approximately 230,000 people, with 25% estimated to be children under-five years. The area is predominantly Islamic with two main ethnic groups—Hausa and Fulani, living in mostly rural communities [12]. At study inception, Kiyawa LGA had 33 government health facilities, made up of primary health centres (PHCs), basic health centres and health posts, and a further two private primary care facilities and private pharmacy shops. Children who meet WHO criteria for referral should be sent to PHCs or hospital care at Dutse General or Rasheed Shekoni Teaching hospital, both located in the state capital, Dutse, in the neighbouring LGA (Fig. 2). Maternal and child health services are provided free at selected facilities, but out-of-pocket costs still pose a financial barrier to access [4]. Kiyawa LGA was selected through discussion between Save the Children Nigeria, the State Ministry of Health (SMOH) and the Jigawa State Government, using pre-defined criteria (e.g. mortality burden, road access).

### Design

This is a pragmatic community-based cRCT, following a formative phase of intervention development, concept testing and community engagement. Impact against the primary outcome will be evaluated using an open prospective cohort, recording vital events (pregnancies, births and under-five child deaths) amongst a random sample of women on a rolling 4-monthly basis. Secondary outcomes will be recorded during cross-sectional surveys conducted at baseline (January–March 2021) and end line (July–September 2022) (Fig. 1). The same random sample of compounds, and all eligible women and children residing in these compounds, will be included in both surveys and prospective cohort.

Clusters are defined as the geographical catchment area of the 33 government primary care facilities. Clusters range in population size from approximately 1680 to 24,200 (median 5840). All villages in Kiyawa LGA will be included in the study (*n* = 425 enumerated villages); however, the Kiyawa PHC cluster will be excluded from the impact evaluation as this facility is in a peri-urban area and provides a level of inpatient care—unlike the other primary facilities. Villages are made up of compounds, which are defined as the group of structures (including ‘households’) where extended families live and have shared resources.

### Population

The target population for the impact evaluation is women of childbearing age, defined as 16–49 years inclusive, and children aged 0–59 months inclusive, who reside permanently in Kiyawa LGA. Women and children will be considered lost to follow-up if they migrate, are temporarily unavailable, or consent is withdrawn. Children of women who are lost to follow-up, or die during the study period, will still be included if they continue to reside in the same compound and an eligible caregiver can provide consent. The intervention is open to all community members, with no age or gender restrictions.

### Intervention

The intervention is a locally adapted ‘whole systems strengthening’ package of three evidence-based activities: PLA men’s and women’s groups [8], Partnership Defined Quality Scorecard (PDQS) [13–15] and training, mentorship and provision of basic essential equipment and commodities for child health (Table 1). The details of the community groups and PDQS have been refined through a co-design process, using a modified version of the community conversation methodology. In its original form, the approach has been used across a range of settings in Africa to shift and influence behaviour change in relation to health, including HIV/AIDS [16] and female genital mutilation [17]. Though typically applied as an intervention itself, our reformulation was designed to deepen engagement with communities at scale as part of the concept testing process. Conversations were organised in a similar manner to the PLA groups that will form one component of the intervention, providing an additional layer for exploring the acceptability of this approach prior to implementation. Groups of stakeholders, including men, women and healthcare workers, were brought together through a series of iterative and interactive discussions to explore perceptions of key concepts underpinning the interventions (e.g. challenges related to child health), community relationships with healthcare workers and how key intervention components would work best in their communities, including location and timing for delivery of groups and incentives. Full details of this methodology and results will be published separately.

#### PLA groups with integrated PDQS

Groups of approximately 20–30 men and women in each community will be convened. Group meetings will be held separately for men and women to allow gender norms to be respected, and tailor meetings to different locations and times. Groups are open to all community members, and sharing of group messages will be encouraged; men and women with young children and those currently pregnant will be particularly encouraged to join.

**Fig. 3:**
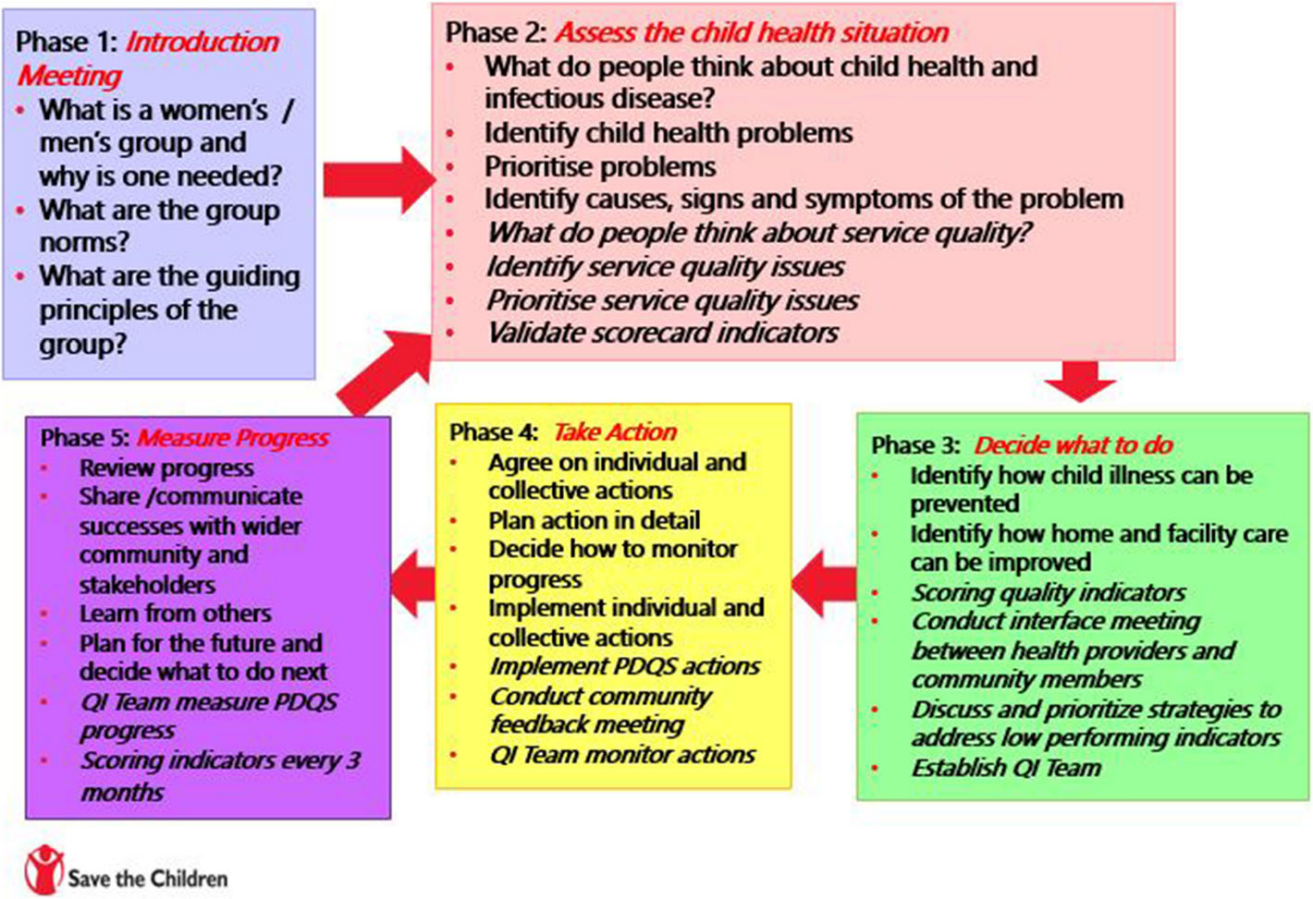
Women’s and men’s group PLA cycle

The groups will work through a four-phase PLA cycle, following an initial introductory session. The phases include the analysis of the situation and prioritisation of child health issues in their communities (phase 1), planning actions to tackle these issues (phase 2), implementing the selected strategies (phase 3) and collective self-evaluation (phase 4) (Fig. 3). The PDQS process focussed specifically on healthcare service quality issues, with group members and healthcare workers joining together to mutually agree on quality indicators and joint actions. PDQS will be incorporated into all 4 phases of the PLA cycle, where healthcare workers will attend men’s and women’s groups. Each phase can involve multiple physical meetings, decided on by group members (see the “Ethical considerations” section for details on COVID-19 mitigations). The completion of one PLA cycle is anticipated to take 4–5 months, and groups will repeat the cycle multiple times over the 18-month intervention period.

We plan 340 groups to ensure the recommended coverage of one group per 500 population [6]. Groups will be led by 170 trained community health volunteers (CHVs), provided with a stipend. CHVs will facilitate an average of two groups, with 12 to 28 groups per cluster, depending on the population. CHVs will be recruited from the local communities, meeting the following criteria: previous experience in community mobilisation, basic knowledge of child health, fluent in Hausa, minimum basic primary education and resident in Kiyawa LGA. Where possible, facilitators will be recruited from those working as Volunteer Community Mobilizers (VCM), Team Supervisors or other community health workers. They will receive a 5-day training, covering the PLA approach, their roles and responsibilities, participatory skills development using observation and feedback, use of the flipbook job aid and collaboration with key community stakeholders. Following training, a questionnaire assessment will be administered, and only those showing adequate competencies in networking, negotiation and mediation, and basic child health knowledge will be retained. CHVs will be supervised by Save the Children study staff based in Dutse, in collaboration with the SMOH, Kiyawa LGA staff and health facility staff.

#### Health system strengthening

The staff at health posts, basic health centres and PHCs within the intervention clusters will receive a package of trainings to support child health, including iCCM for community health workers, IMCI for facility staff, nutrition management and vaccination supply chain management (Table 2). Training will be delivered at the start of the intervention period and then followed up with mentorship and supervision. Training and supervision will be delivered by Save the Children Nigeria in collaboration with the SMOH. A post-training ‘start-up kit’ will be provided, including basic essential equipment (e.g. respiratory rate timers, thermometers, MUAC tape) and guideline booklets and charts.

**Table 2:** Description of health system strengthening components

Training	Participants	Training structure	Trainers	On-going support	Donated supplies and equipment
Integrated Community Case Management (iCCM) [18]	3 staff from the 17 intervention facilities, including junior CHEWs and CHIPS volunteers	6-day training, including classroom (presentations and clinical scenarios) and clinical practice sessions	Certified iCCM trainer	6-weekly* supervision visits by training facilitators and Save the Children staff	Training modules and M&E tools Job aids and chart booklets Start-up kit, including RR timer, ORS + zinc, paediatric ambu bag, MUAC tapes, thermometer
Integrated Management of Childhood Illness (IMCI) [19]	50 staff from 17 intervention facilities, including CHEWs, CHOs, nurses and doctors	6-day training, including classroom (presentations and clinical scenarios) and clinical practice sessions	Certified IMCI trainer		
Immunisation “Reaching Every District”	40 staff from 17 intervention facilities, including CHEWs, CHOs, nurses and doctors	2–3-day training, covering management, planning, data, supervision, engagement and outreach services	Certified Immunisation trainer	Financial and logistics outreach support	Vaccine carrier bags 3 electric and 2 solar refrigerators M&E tools, charts and guidelines
Nutrition–Infant and Young Child Feeding (IYCF)	50 CHO, junior CHEWs and CHEWs, and nutrition focal persons	3-day training, including classroom and practical learning	State Ministry of Health	Provision of IYCF corner, supporting food demonstrations, and supportive supervision.	Training modules and M&E tools Job aids and chart booklets MUAC tapes Plumpy nut
Pulse oximetry and oxygen therapy	25 staff at 2 referral hospitals 6 staff at 3 PHCs including CHEWs, CHOs, nurses and doctors	3-day training, including classroom and practical learning	Oxygen for Life Initiative	Annual biomedical engineer equipment audit Bi-monthly* mentorship visits by Save the Children staff	12 Lifebox pulse oximeters with universal and paediatric clip probes 8 oxygen concentrators 40 oxygen cylinders

*This schedule applies to the first 6 months post-training, after which the supervision and mentorship plan will be reviewed

*CHEW* community health extension worker, *CHO* community health officer, *CHIPS* Community Health Influencers Promoters and Services, *RR* respiratory rate, *ORS* oral rehydration salt, *MUAC* mid-upper arm circumference, *PHC* primary health centre

### Control

All clusters, including control, will have access to a child health radio programme throughout the project period (Fig. 1). The radio programme consists of weekly dramas, covering prevention, recognition and care-seeking for common childhood diseases, including pneumonia. There will be a monthly call-in session, where members of the public can ask questions and share experiences. The radio station is accessible across the whole study area.

The two referral hospitals located in Dutse LGA (Fig. 2), and three PHCs in Kiyawa LGA will be supported with oxygen and pulse oximetry, to ensure minimum standard of care is available for children meeting WHO IMCI referral criteria in intervention and control clusters.

### Outcome

The primary outcome is under-five mortality. This is defined as the number of children aged 7 days to 59 months reported to have died over a 12-month period who resided in sampled compounds (numerator), divided by the total number of children aged 7 days to 59 months who reside in sampled compounds (denominator). Deaths in the first week of life are excluded due to the challenges of accurate reporting of stillbirths and early neonatal deaths [20], and we do not expect the intervention to impact perinatal causes. Secondary outcomes are detailed in Table 3 and are based on the INSPIRING Programme Theory of Change.

**Table 3:** Primary and secondary outcomes

Primary outcome	Definition
Under-five mortality rate	The number of confirmed* deaths amongst children aged 7 days–59 months, per 1000 livebirths reported by women in the cohort, in the last 12 months
**Secondary outcomes**	
Suspected pneumonia mortality rate	The number of confirmed* deaths classified by InterVA-5 as primarily attributable to acute respiratory infections amongst children aged 7 days–59 months, per 1000 livebirths reported by women in the cohort, in the last 12 months [21]
Pneumonia point prevalence	The proportion of children clinically assessed on the day of the survey who meet 2014 WHO IMCI definition for pneumonia or severe pneumonia, including hypoxemia [22, 46]
Women’s wellbeing	The mean Short Warwick-Edinburgh Mental Wellbeing Scale score amongst women aged 16–49 years [23]
*Treat*	
Knowledge of pneumonia	The proportion of women aged 16–49 who can name both fast and difficulty breathing as signs of pneumonia
Care-seeking for childhood illnesses	The proportion of caregivers who self-reported visiting a formal healthcare provider (including primary, secondary, private or government facilities) within 48 h of illness recognition, amongst those who reported their child has been sick in the previous 2 weeks
*Protect*	
Exclusive breastfeeding	The proportion of children aged 0–6 months whose caregiver self-reports exclusively breastfeeding
Vitamin A	The proportion of children aged 18–59 months with 2 doses of vitamin A
*Prevent*	
Vaccine coverage	The proportion of children aged 18–59 months with complete vaccine coverage according to the Nigerian Childhood Vaccination programme [24]**
Handwashing with soap	The proportion of households who self-report access to soap and water in their home for handwashing
Household air pollution	The proportion of households who self-report cooking indoors with wood/charcoal or dried grass

*Deaths which are verified by a field supervisor during a verbal autopsy visit

**Includes 1 dose BCG, 4 doses oral polio vaccine, 2 doses rotavirus vaccine, 3 doses DPT-Penta, 3 doses PCV-10 and 2 doses measles

### Randomisation

The 33 clusters will be randomised using a 1:1 ratio between intervention and control. Randomisation will be done publicly, with representatives from the LGA and all the wards, following community consent for the study and the randomisation process. The names of the health facility clusters will be written on pieces of paper, folded and placed in a container by study staff. A community representative will then pull the folded pieces of paper out of the container one by one, in front of community witnesses. The first 17 will be assigned to the intervention, and the final 16 will be assigned to control. Due to the nature of the intervention, communities will not be blinded to their status; however, we will not inform data collectors of the intervention status of villages or that they work across intervention and control clusters, and investigators will be blinded during primary analysis.

### Sampling

During the formative phase, we conducted a community mapping exercise to create a sampling frame, enumerating all villages and the total number of compounds. We will sample proportionately to cluster size, with a minimum of 50 compounds per cluster. Simple random sampling will be used to generate a list of compound numbers within each cluster. During the baseline survey, data collectors will use an Expanded Programme of Immunization approach [25] to number all the compounds in a village, following these steps: (1) locating the centre of the village with support from a community gate keeper, (2) spinning a pen and walking in the direction it points, (3) numbering the first compound encountered as 1 and (4) walking in a clockwise direction until all compounds are numbered. They will then recruit the compounds according to the generated random sample. The same compounds will be included in all subsequent surveys, and GPS coordinates will be taken to facilitate follow-ups. All eligible women, and children under-five years of age who are under their direct care, residing within sampled compounds will be recruited.

### Sample size

The under-five mortality rate in Jigawa was reported as 192/1000 livebirths in 2017 [26], and deaths in the first week of life may reasonably account for 15% of the total under-five deaths. More recently, a participatory radio programme on child health and COVID-19 was delivered across the LGA (June–December 2020) and vaccine coverage has increased (unpublished data), which we assume to have reduced mortality. We therefore used a conservative baseline mortality of 100 deaths amongst children aged 7 days to 59 months per 1000 livebirths. With the following parameters, we will have a minimum detectable effect size of 30% [6]: (a) 380 children per cluster, (b) 80% power, (c) 5% significance, (d) intra-cluster correlation of 0.007 [27] and (e) coefficient of variance of cluster size of 0.74. We assume on average there are three children under-five per compound (i.e. 127 compounds per cluster), and therefore, we will sample 4480 compounds overall to allow for ~ 10% loss to follow-up. The sample size was calculated using Stata’s *-clustersampsi-* command [28]. We will review these assumptions following the baseline survey.

### Data collection

Data collection will be done by 20 female clinical data collectors recruited from Jigawa, with two field supervisors. They will work in teams of 3–4, accompanied by a lay data collector to support community mapping. A sub-set of data collectors will be retained between surveys to conduct prospective vital event reporting. Data will be collected using a custom-built CommCare application on Android tablets. The forms will have in-built skip patterns and cleaning rules to minimise data entry errors. Data will be uploaded daily to a central secure server, and participant ID and data cleaning checks will be conducted continually during the data collection period. Data collectors will be trained using a hybrid approach, with a mix of practical sessions, at-home assignments and online webinars over a 2-week period. This will be followed by a field pilot in a neighbouring LGA and mock interviews with a group of women in the community who took part in questionnaire co-design discussions.

#### Baseline and endline surveys

The baseline and endline surveys are planned to take 3 months, with approximately 70 compounds completed each day. Three respondent types will be interviewed in each compound: (1) head of compound, (2) head of households and (3) eligible women. The head, or most senior member of the compound present at the time of the survey, interview will include compound membership, structure and construction; shared compound and individual household assets and income; and community cohesion. Household heads will be asked about household membership and asset ownership. Women will be asked about themselves, and then about each of the children under-five who are in their direct care. Questions include recent birth history, demographics and social status, knowledge of pneumonia, recent illness episodes and care-seeking, vaccination status, feeding practices, smoke exposure, community cohesion and wellbeing (using the Short Warwick-Edinburgh Mental Wellbeing Scale [23]). Questions on pneumonia knowledge and recent illness will use multi-media (i.e. videos and sounds) to provide examples of key signs and symptoms. In the endline survey, additional questions will be asked about intervention exposure and engagement.

A clinical screening for malnutrition and pneumonia will be conducted on all children aged 0–59 months who are present in the compound at the time of the survey. The assessment will follow the WHO’s 2014 IMCI guidelines [19﻿], enhanced with pulse oximetry using the Lifebox pulse oximeter (Acare Technology, New Taipei City, Taiwan). If a child is found with pneumonia, the data collector will inform the caregiver and advise them to seek care and facilitate referral in cases of severe illness.

#### Prospective vital event reporting

Following recruitment, the same compounds will be visited on a four-monthly rolling basis. All previously registered women will be asked about their current pregnancy status, the outcome of previously reported and completed pregnancies and the vital status of all registered children. Any new children under-five will be registered and included in the cohort. Verbal autopsies (VAs) will be conducted for all deaths in children under-five, including those reported in the prior 12 months from birth histories. This time period was chosen to minimise recall bias, but ensure a culturally appropriate period of time has passed since the death. We will use the COVID-19 adapted WHO 2016 VA tool and include locally adapted social autopsy questions which explore care-seeking pathways [29, 30].

### Analysis

The primary analysis will be intention-to-treat, comparing under-five mortality between intervention and control clusters over a 12-month period (July 2021–June 2022), using mixed-effects logistic regression, with fixed effects for the trial arm (intervention or control), and random effects to adjust for compound-level clustering and trial cluster. Data from the first 6 months of intervention implementation (January–June 2021) will be considered as a baseline period, before any intervention effect would be expected. Any imbalances in baseline characteristics between arms will be presented to the Trial Steering Committee (TSC), who will advise on whether the primary analysis should be adjusted for these. We will conduct a complete case analysis, where only children with confirmed survival outcomes will be included; a sensitivity analysis using multiple imputation will be done, if outcome data is found to be missing at random.

Secondary analyses, using the same mixed-effects model approach, will explore differences in pneumonia prevalence, coverage of prevent and protect indicators, knowledge and care-seeking between intervention and control clusters (Table 3). Changes in pneumonia prevalence, knowledge and care-seeking between baseline and endline surveys will be described and compared using chi-squared tests.

## Process evaluation

We will also conduct a concurrent mixed-methods process evaluation (Table 1, Additional file 1), in line with UK Medical Research Council guidelines to describe intervention implementation and develop the theory of how the intervention can affect health outcomes in this context [31]. The process evaluation will start before the intervention period, from June 2020, and continue throughout the trial period.

### Qualitative

The qualitative aspect will take an ethnographic approach [32], to describe how, why, where, when and for whom (under what conditions) the intervention works. Our approach enables us to develop understandings of outcomes within the social context and build theory grounded in real-world settings [32]. It ensures that the theory of change and outcomes identified through the intervention can be explored as part of an iterative analysis that remains anchored to the daily realities of implementation, delivery and uptake of the intervention. The ethnography will operate at three levels: household, health services and intervention. Each level will include a blend of participant observation, longitudinal in-depth interviews and focus group discussions. At the household level, we will follow a sample of 30 randomly selected households, stratified to reflect intervention (*n* = 3) and control (*n* = 3) clusters. Up to five women from each household will be invited to participate. PLA groups associated to these clusters will also be observed through participant observation, to explore live dynamics of groups. Health facilities in each cluster will also be observed monthly across the intervention.

Data will be collected by a minimum of three female Nigerian research assistants. All field notes, interviews and discussions will be transcribed and translated into English for analysis. Monthly analysis meetings between the data collectors and RAB will be done to promote reflexive practice. These meetings feed into our analysis approach, which applies Bren Neale (2019) conceptual scaffolding approach to analysis of qualitative longitudinal data [33]. We use a diversity of methods, within an iterative process of visiting the data across the course of collection prior to the deep analysis period at the end of the project. Different types of ethnographic data will be analysed using different methods, working across themes and time to understand complex relationships between knowledge and behaviour. Longitudinal interviews will be analysed using a case analysis method and framework analysis [34]. FGD and observational data will be analysed using thematic analysis [35].

### Quantitative

The quantitative component involves quarterly extraction of clinical case notes and ward admission logs from all 33 facilities in Kiyawa LGA and the two secondary referral hospitals in Dutse LGA. This will allow us to monitor changes in both clinical practice, diagnosis, cases of suspected pneumonia and inpatient case-fatality rates during the study period. An annual equipment audit will be conducted by a qualified biomedical engineer, to check the availability of functional medical grade oxygen in Jigawa (i.e. minimum standard of care for severe pneumonia cases). CHV group facilitators will keep meeting logs, recording the number of meetings, meeting attenders and their demographics, and key action plans and decisions made by groups. These data will be summarised using means, medians, proportions and 95% confidence intervals to describe intervention reach and the different strategies employed by groups.

## Economic evaluation

The economic evaluation will determine the cost-effectiveness of the intervention package relative to current and future spending (opportunity costs and affordability) [36–38]. This is crucial considering the current paucity of economic evaluation evidence for interventions to tackle childhood infectious diseases and pneumonia and will be important to understand in the context of the COVID-19 pandemic. We will conduct a prospective costing of the interventions which will include financial (i.e. capital set-up and recurrent expenditures) [39] and economic costs, including time, and be based on the Ministry of Health provider perspective. We will also consider the household perspective via surveys of financial and opportunity costs of care-seeking to caregivers.

## Management and oversight

Save the Children Nigeria will be responsible for the coordination and implementation of the intervention, working closely with the State Ministry of Health. University College Hospital Ibadan, in collaboration with University College London, Karolinska Institutet, Melbourne University and Johns Hopkins University, will be responsible for the impact, process and economic evaluation. All co-investigators will have access to the final trial dataset. Save the Children UK, GSK and GSK Nigeria will be involved in overall project management. The Project Management Board will meet monthly and include representatives from all partners.

A TSC will be convened for the study, with a plan to meet a minimum of three times (protocol review, mid-point, final analysis), and in between if major ethical or design issues arise. The TSC will be responsible for providing independent and external oversight of the trial conduct, including reviewing processes, progress, participant safety and protocol amendments. The TSC will comprise subject-specific experts and will include the following disciplines: statistician, clinician, social scientist and epidemiologist. We are not planning to conduct an interim analysis given the short intervention period and do not have stop/start rules in place. We will not convene a separate Data Monitoring Committee; data monitoring and cleaning will be done continuously and overseen by the principal investigators, with cleaning files and logs shared with co-investigators and field staff. The trial is sponsored by University College London, Institute for Global Health (30 Guildford Street, London, WC1N 1EH, UK).

## Ethical considerations

We have two key ethical concerns. First is the study staff responsibility to act when faced with participants in need. Specifically, if a child is diagnosed with a severe illness according to WHO IMCI criteria during compound visits, they should be referred for inpatient care. In such cases, we will support the family in finding transportation and inform the caregiver of the diagnosis. Furthermore, we are aware that in our in-depth engagement with 30 compounds over the life of the study as part of the process evaluation, we may identify safeguarding issues, interpersonal violence, health emergencies and challenges in accessing basic livelihood needs. We have a detailed protocol that data collectors will be trained on, to escalate immediately through local partners, so that any extraordinary needs can be addressed quickly.

Secondly, the nature of PLA groups could increase participants’ risks of transmitting COVID-19 if there is on-going community transmission. Implementation will only begin following a local risk assessment, approved by the Project Management Board. The assessment will be based on reported local and national numbers of COVID-19 cases and deaths and in accordance with local government advice. As an additional precaution, all facilitators and study staff will be provided with face masks, hand sanitizer and training on physical distancing procedures. Community sensitisation about COVID-19 was conducted through a participatory radio programme from June to December 2020, and study staff will continue to promote infection prevention and control messages. Aside from the COVID-19 risks, we do not anticipate any other serious negative impacts to participants taking part, and the intervention package could provide benefit to individuals and wider communities.

### Equipoise

Given the different components of the intervention package have previously shown impact on child health outcomes, equipoise needs to be justified. Firstly, combining the three intervention elements into a single complex intervention package is novel. Secondly, implementation of PLA groups in the context of Jigawa, with conservative gender norms and complex compound relationship structures, may pose unique challenges. Finally, the public health messages from the COVID-19 pandemic around physical distancing may have changed how communities meet and interact. We felt all of these factors provide sufficient uncertainty to justify the cRCT design.

### Consent

Informed verbal or written consent will be sought from all participants for interviews, focus group discussions and household surveys. Participants will be reimbursed for any transport costs incurred when taking part in the study, and given a small token of appreciation (e.g. soap, face mask) following interviews. Consent for case note reviews from primary and secondary care will be sought from facility managers.

## Engagement and dissemination

Engagement with key stakeholders, including healthcare workers, communities and both State and LGA Ministry of Health officials, will be done continually during the project. The overall study aims and focus were developed through round table discussions with project partners, and representatives from the SMOH before starting the trial. Meetings with community members to co-design intervention delivery were done during the formative phase, and multiple meetings with community representatives to inform them of the study, gain consent and promote recruitment and engagement have been done.

Dissemination events will be held with the SMoH and with communities during project organised meetings within communities at the end of the project. These events will share the findings, present plans for future implementation and discuss handover. However, regular meetings will be held as and when needed to share important findings, discuss interpretation and report on progress. We will publish the main impact results, process evaluation and economic evaluation results as academic publications in open access journals.

## Discussion

This cRCT aims to provide robust impact and cost-effectiveness evidence for whole systems—strengthening intervention to improve child health, with a focus on pneumonia, in a high mortality setting.

Our main design challenge was the power calculation. The most recent mortality estimate for Jigawa is from the 2018 Demographic Health Survey [12]. However, the Jigawa State Government has implemented several child health programmes in recent years, including a community nutrition programme (‘Masaki’) covering 10 communities in Kiyawa LGA, increasing vaccine coverage, and a Health Insurance Scheme. This may be further confounded by the recently launched Basic Healthcare Provision Fund, which applies to both intervention and control clusters during the trial period.

The COVID-19 pandemic introduces further uncertainty in the baseline mortality estimate. Modelled reductions in access to care, vaccination coverage and increased malnutrition due to disrupted global supply chains would halt, or reverse mortality gains [40, 41]. Nigeria went into recession in 2020, which could also negatively affect child health and survival. Conversely, empirical data from high-income settings has demonstrated reductions in acute paediatric respiratory hospitalisations resulting from COVID-19 public health measures [42–44]. Investment in water and sanitation, a focus on handwashing and efforts to strengthen oxygen systems in response to COVID-19 should all improve child survival.

A 30% intervention effect on mortality is reasonable, given previous PLA trials and clinical interventions to improve pneumonia case management [6, 45]. However, we risk being under-powered if under-five mortality has declined more rapidly than we presumed, or we are unable to reliably record death events. Community-reported mortality surveillance poses challenges and can suffer from under-reporting and misclassification of stillbirths and early neonatal deaths [21, 22, 24]. In order to mitigate this concern, we re-designed the study from protocol version 1.0, which relied solely on retrospective death reporting through cross-sectional surveys, to incorporate prospective vital events surveillance. We also used the ethnographic platform to adapt and pilot test survey tools, exploring how child deaths are discussed by communities in this context.

## Trial status

Protocol version 2.3 (10 January 2021). As of the 15th September 2021, the baseline survey has been completed and prospective vital signs surveys started. PLA groups have been set up and are running, and some components of health system strengthening implemented. The protocol history is as follows: v1.0 (20 August 2019); v1.1 (23 August 2019) process evaluation incorporated; v1.2 (2 September 2019) minor language edits; v1.3 (3 September 2019) ethical approval added; v2.0 (4 June 2020) COVID-19 adaptation included; v2.1 (14 June 2020) COVID-19 qualitative data collection procedures; v2.2 (20 October 2020) prospective vital events reporting added following TSC recommendation; v2.3 (10 January 2021) sample size calculation updated to include cluster size variance.

## Supplementary Information

**Additional file 1.** Process evaluation indicators

## Data Availability

Any data required to support the protocol can be supplied on request. A fully anonymised limited trial dataset will be uploaded to an open access data repository alongside the primary impact result publication. Anonymised process and economic data will be available upon reasonable request after a 12-month period, to allow the project team time to analyse and publish these data.

## Abbreviations

CHV: Community health volunteer
cRCT: Cluster randomised controlled trial
FGD: Focus group discussion
iCCM: Integrated Community Case Management
IMCI: Integrated Management of Childhood Illnesses
INSPIRING: Integrated Sustainable childhood Pneumonia and Infectious disease in children Reduction in Nigeria
LGA: Local Government Authority
LMIC: Low- and middle-income country
PDQS: Partnership Defined Quality Scorecard
PHC: Primary health centre
PLA: Participatory learning and action
SMOH: State Ministry of Health
TSC: Trial Steering Committee
WHO: World Health Organization
