# Supplementary material for: Integrated Sustainable childhood Pneumonia and Infectious disease Reduction in Nigeria (INSPIRING) through whole system strengthening in Jigawa, Nigeria: study protocol for a cluster randomised controlled trial
Source: Trials. 2022 Jan 31;23:95. doi: 10.1186/s13063-021-05859-5 (PMC8802253; doi:10.1186/s13063-021-05859-5)
Supplement: Supplementary file 1 — Additional file 1. Process evaluation indicators [file 13063_2021_5859_MOESM1_ESM.pdf]

Process Indicators

	Indicator	Frequency of data collection	Person collecting	Source of data	Tool required	Data type
Intervention indicators (community groups)						
Intervention fidelity	Training fidelity (facilitator)	Once	Jigawa UCH manager	Observation	Checklist	Mixed
	Number of sessions conducted	Monthly	Facilitator	Register	Log book	Quantitative
	Content of sessions	Monthly	Facilitator	Register	Log book	Quantitative
	Participatory-ness of sessions	Monthly	Facilitator	Register	Log book	Mixed
	Supervision visits conducted	Monthly	STC supervisor	Work plans	Work plan	Quantitative
Intervention reach	Number of attendees	Monthly	Facilitator	Register	Log book	Quantitative
	Profile of attendees	Monthly	Facilitator	Register	Log book	Quantitative
	Distance travelled	Monthly	Facilitator	Register	Log book	Quantitative
Facilitator characteristics	Number trained to competency	Once	STC trainer	Observation	Checklist	Quantitative
	Number retained	Once	STC trainer	HR records	Questionnaire	Quantitative
	Facilitator profiles	Once	STC trainer	HR records	Questionnaire	Quantitative
	Intervention strategies	Once	Facilitator	Register	Log book	Quantitative
	Trainer/supervisor perceptions	Midline/endline	UCH data collectors	Interviews	Topic guide	Qualitative
	Facilitator perceptions	Midline/endline	UCH data collectors	Group discussions	Topic guide	Qualitative
Intervention indicators (community-facility link)						
Intervention fidelity	Training fidelity (facilitator)	Once	Field manager	Observation	Checklist	Mixed
	Number of sessions conducted	Monthly	Facilitator	Register	Log book	Quantitative
	Content of sessions	Monthly	Facilitator	Register	Log book	Quantitative
	Action points developed	Monthly	Facilitator	Register	Log book	Quantitative
	Action points delivered	Monthly	Facilitator	Register	Log book	Quantitative
Intervention reach	Number of attendees	Monthly	Facilitator	Register	Log book	Quantitative
	Profile of attendees	Monthly	Facilitator	Register	Log book	Quantitative
	Distance travelled	Monthly	Facilitator	Register	Log book	Quantitative
Facilitator characteristics	Number trained to competency	Once	STC trainer	Observation	Checklist	Quantitative
	Number retained	Once	STC trainer	HR records	Questionnaire	Quantitative
	Facilitator profiles	Once	STC trainer	HR records	Questionnaire	Quantitative

	Intervention strategies	Once	Facilitator	Register	Log book	Quantitative
	Trainer/supervisor perceptions	Midline/endline	UCH data collectors	Interviews	Topic guide	Qualitative
	Facilitator perceptions	Midline/endline	UCH data collectors	Group discussions	Topic guide	Qualitative
Intervention indicators (health systems)						
Intervention fidelity	Training fidelity	Once	Jigawa STC manager	Observation	Checklist	Mixed
	Pre/Post testing	Once	STC trainer	Survey	Questionnaire	Quantitative
	Supervision reports	Monthly	STC supervisor	Observation	Checklist	Qualitative
	Supervision visits conducted	Monthly	STC supervisor	Work plans	Work plans	Quantitative
	Training coverage over time	Biannual	STC trainer	Observation	Checklist	Quantitative
	Oxygen system functionality	Base/mid/endline	UCH data collectors	Audit	Questionnaire	Quantitative
	Oximeter functionality	Base/mid/endline	UCH data collectors	Audit	Questionnaire	Quantitative
Facilitator characteristics	Trainer/supervisor perceptions	Midline/endline	UCH data collectors	Interviews	Topic guide	Qualitative
	Provider perceptions	Midline/endline	UCH data collectors	Group discussions	Topic guide	Qualitative
Context	Drug stock audits	Quarterly	STC supervisor	Observation	Questionnaire	Quantitative
	Supply chain audits	Quarterly	STC supervisor	Observation	Questionnaire	Quantitative
	HMIS audits	Quarterly	STC supervisor	Observation	Questionnaire	Quantitative
Clinical indicators						
	Total case numbers	Quarterly	Jigawa UCH manager	Facility register	Extraction tool	Quantitative
	Proportion pneumonia diagnoses	Quarterly	Jigawa UCH manager	Facility register	Extraction tool	Quantitative
	Correct IMCI assessment	Quarterly	Jigawa UCH manager	Facility register	Extraction tool	Quantitative
	Correct IMCI diagnosis	Quarterly	Jigawa UCH manager	Facility register	Extraction tool	Quantitative
	Correct treatment decision	Quarterly	Jigawa UCH manager	Facility register	Extraction tool	Quantitative
	Correct referral decision	Quarterly	Jigawa UCH manager	Facility register	Extraction tool	Quantitative
	Oxygen treatment	Base/mid/endline	Data collector	Household interviews	Questionnaire	Quantitative
	Secondary care CFR	Quarterly	Jigawa UCH manager	Facility register	Extraction tool	Quantitative
	Pneumonia point prevalence	Base/mid/endline	Data collector	Household interviews	Questionnaire	Quantitative
	Pneumonia 2-week prevalence	Base/mid/endline	Data collector	Household interviews	Questionnaire	Quantitative
	Malnutrition	Base/mid/endline	Data collector	Household interviews	Questionnaire	Quantitative
Community behaviours						
	Immunisation	Base/mid/endline	Data collector	Household interviews	Questionnaire	Quantitative

	Exclusive breastfeeding	Base/mid/endline	Data collector	Household interviews	Questionnaire	Quantitative
	Cooking fuel	Base/mid/endline	Data collector	Household interviews	Questionnaire	Quantitative
	Water and Sanitation	Base/mid/endline	Data collector	Household interviews	Questionnaire	Quantitative
	Vitamin A	Base/mid/endline	Data collector	Household interviews	Questionnaire	Quantitative
	Soap for handwashing	Base/mid/endline	Data collector	Household interviews	Questionnaire	Quantitative
	Location of first seeking care	Base/mid/endline	Data collector	Household interviews	Questionnaire	Quantitative
	Any care seeking	Base/mid/endline	Data collector	Household interviews	Questionnaire	Quantitative
	Smoking exposure	Base/mid/endline	Data collector	Household interviews	Questionnaire	Quantitative
	TB exposure	Base/mid/endline	Data collector	Household interviews	Questionnaire	Quantitative

Economic Indicators

Indicator		Frequency of data collection	Person collecting	Source of data	Tool required	Data type
Provider side (community group)						
Start-up costs	Training facilitators	Baseline	STC administration	STC accounts	Budget tool	Quantitative
	Facilitator recruitment	Baseline	STC administration	STC accounts	Budget tool	Quantitative
	Community entry	Baseline	STC administration	STC accounts	Budget tool	Quantitative
Implementation costs	Equipment	Endline	STC administration	STC accounts	Budget tool	Quantitative
	Facilitators allowances	Endline	STC administration	STC accounts	Budget tool	Quantitative
	Supervisor salaries	Endline	STC administration	STC accounts	Budget tool	Quantitative
	Supervision costs	Endline	STC administration	STC accounts	Budget tool	Quantitative
	Overhead/infrastructures	Endline	STC administration	STC accounts	Budget tool	Quantitative
Time cost	Travel time and cost	Monthly	Facilitators	Meeting registers	Log-book	Quantitative
	Meeting duration	Monthly	Facilitators	Meeting registers	Log-book	Quantitative
Patient side (community meetings)						
Meeting attendance	Travel time and cost	Mid/endline	UCH data collector	Household survey	Questionnaire	Quantitative
	Meeting duration	Mid/endline	UCH data collector	Household survey	Questionnaire	Quantitative
	Opportunity costs	Mid/endline	UCH data collector	Household survey	Questionnaire	Quantitative

Group activities	Time spent on group activity	Mid/endline	UCH data collector	Household survey	Questionnaire	Quantitative
	Money spent on group activity	Mid/endline	UCH data collector	Household survey	Questionnaire	Quantitative
	Income generating activities	Once	UCH data collector	One-off survey	Questionnaire	Quantitative
Provider side (community-facility link)						
Start-up costs	Training facilitators	Baseline	STC administration	STC accounts	Budget tool	Quantitative
	Facilitator recruitment	Baseline	STC administration	STC accounts	Budget tool	Quantitative
	Community entry	Baseline	STC administration	STC accounts	Budget tool	Quantitative
Implementation costs	Facilitators allowances	Endline	STC administration	STC accounts	Budget tool	Quantitative
	Supervisor salaries	Endline	STC administration	STC accounts	Budget tool	Quantitative
	Supervision costs	Endline	STC administration	STC accounts	Budget tool	Quantitative
	Overhead/infrastructures	Endline	STC administration	STC accounts	Budget tool	Quantitative
Time cost	Travel time and cost	Monthly	Facilitators	Meeting registers	Log-book	Quantitative
	Meeting duration	Monthly	Facilitators	Meeting registers	Log-book	Quantitative
Patient side (community-facility link)						
Meeting attendance	Travel time and cost	Mid/endline	UCH data collector	Household survey	Questionnaire	Quantitative
	Meeting duration	Mid/endline	UCH data collector	Household survey	Questionnaire	Quantitative
	Opportunity costs	Mid/endline	UCH data collector	Household survey	Questionnaire	Quantitative
Group activities	Time spent on group activity	Mid/endline	UCH data collector	Household survey	Questionnaire	Quantitative
	Money spent on group activity	Mid/endline	UCH data collector	Household survey	Questionnaire	Quantitative
Provider side (PPE/IPC and oxygen strengthening)						
Start-up costs	IPC/PPE training	Endline	STC administration	STC accounts	Budget tool	Quantitative
	IPC/PPE equipment	Endline	STC administration	STC accounts	Budget tool	Quantitative
	Pulse oximeters	Endline	UCL	UCL accounts	Invoices	Quantitative
	Oxygen concentrators	Endline	UCL	UCL accounts	Invoices	Quantitative
	Pulse oximetry training	Endline	Oxygen for Life	OLI accounts	Invoices	Quantitative
	Oxygen training	Endline	Oxygen for Life	OLI accounts	Invoices	Quantitative
Implementation costs	Supervision	Endline	STC administration	STC accounts	Budget tool	Quantitative
	Salaries	Endline	STC administration	STC accounts	Budget tool	Quantitative
	Maintenance	Endline	STC administration	STC accounts	Budget tool	Quantitative
Time cost	Time use	Baseline/endline	UCH data collector	Observation	Checklist	Quantitative

Patient side (Health system strengthening)						
	Cost to patient	Baseline/endline	UCH data collector	Household survey	Questionnaire	Quantitative
	Caregiver motivations	Baseline/endline	UCH data collector	Household survey	Questionnaire	Quantitative
	Time taken to seek care	Baseline/endline	UCH data collector	Household survey	Questionnaire	Quantitative

STC: Save the Children; UCH: University College Hospital Ibadan; OLI: Oxygen for Life Initiative; IPC: Infection prevention and control; PPE: personal protective equipment
